# MRI features of craniopharyngiomas in different age groups and pathological subtypes

**DOI:** 10.3389/fonc.2026.1750802

**Published:** 2026-05-05

**Authors:** Weijian Wang, Wenjing Li, Xinyu Wang, Yichen Guo, Longyao Ma, Bohui Mei, Mengzhe Zhang, Hongwei Zheng, Kaixin Li, Mengzhu Wang, Ankang Gao, Yong Zhang

**Affiliations:** 1 Department of Magnetic Resonance Imaging, The First Affiliated Hospital of Zhengzhou University, Zhengzhou, Henan, China; 2 MR Research Collaboration, Siemens Healthineers Ltd., Beijing, China

**Keywords:** adamantinomatous craniopharyngioma, adult, juvenile, magnetic resonance imaging, squamous papillary craniopharyngioma

## Abstract

**Objective:**

To investigate the MRI features of different pathological types in adult and juvenile patients with craniopharyngioma.

**Materials and methods:**

The preoperative MRI images of 68 patients with pathologically confirmed craniopharyngioma were retrospectively analyzed. The tumor location, imaging findings and surrounding tissue involvement were determined, and the tumor volume was measured. The differences of these imaging indicators and pathological types between 48 adult and 20 juvenile groups were compared.

**Results:**

Grouped by age: The proportions of adamantinomatous craniopharyngioma (ACP) in the juvenile group and adult group were 85.0% and 56.3%, respectively, with a significant difference (P = 0.024). The predilection sites of craniopharyngioma differed between the juvenile and adult groups. Tumors in the juvenile group were most frequently located in the intrasellar or suprasellar region (80%), whereas those in the adult group were predominantly suprasellar (60.4%), with a significant difference (P<0.001). The incidence of tumor calcification was 55.0% in the juvenile group and 29.1% in the adult group, with a significant difference (P = 0.044). Grouped by pathological subtype: The mean ages at onset were 27 years for ACP and 43 years for SPCP, with a significant difference (P<0.001). The incidence of cystic degeneration was 95.5% in ACP and 83.3% in SPCP, with a significant difference (P = 0.037). Fluid-fluid levels or sediment were present in 20.5% of ACP, but absent in all SPCP, with a significant difference (P = 0.022). Patients in the non-third ventricular floor invasion group were younger and had a wider age range than those in the third ventricular floor invasion group(P = 0.025).

**Conclusion:**

There is a close correlation between age at onset, pathological subtype, and MRI features of craniopharyngiomas. ACP is more common in juvenile patients, with a higher tendency toward intrasellar/suprasellar location, calcification, and cystic degeneration. In adult patients, lesions are predominantly suprasellar and more frequently accompanied by pituitary involvement. ACP show a higher cystic degeneration rate with characteristic fluid-fluid levels or sediment, which can be used for preoperative pathological differentiation. Meanwhile, invasion of the floor of the third ventricle is more commonly seen in older patients, suggesting that age may assist in evaluating the invasive growth pattern of craniopharyngiomas.

## 1 Introduction

Craniopharyngioma is a common benign intracranial tumor derived from Rathke’s pouch remnants, most frequently occurring in the sellar and parasellar region, with a low propensity for metastasis and distant recurrence. Craniopharyngiomas account for approximately 1% to 3% of all primary intracranial neoplasms in adult populations, whereas in pediatric cohorts, this proportion ranges from 5% to 10% (1). Despite being classified as WHO grade I tumors, craniopharyngiomas often exhibit a clinically aggressive course (2). Craniopharyngioma frequently involves anatomically adjacent structures such as the third ventricle, the hypothalamic-pituitary axis, and the optic nerves/chiasm, leading to dysregulation of hormone secretion—including growth hormone, gonadotropins, thyroid-stimulating hormone, and adrenocorticotropic hormone. Craniopharyngioma is pathologically classified into adamantinomatous craniopharyngioma (ACP) and squamous papillary craniopharyngioma (SPCP). The ACP exhibits a bimodal age distribution, with incidence peaks in children aged 5–15 years and adults aged 45–60 years (3, 4). SPCP predominantly affects adults, with a mean age at diagnosis of 44 ± 15 years (5, 6).

The symptoms of craniopharyngioma are insidious, and the diagnosis is often delayed due to the slow growth of the tumor. Common clinical manifestations of craniopharyngioma include headache, visual impairment, nausea and vomiting, and endocrine deficiency (1). Due to the particularity of anatomical structure, craniopharyngioma often causes hypothalamic invasion (7). Radiotherapy after surgical resection is still considered the gold standard for craniopharyngioma (8). In this paper, we retrospectively analyzed the preoperative MRI data of 68 patients with craniopharyngioma confirmed by surgery and pathology in our hospital and summarized the MRI characteristics of patients with different ages and different pathological types of craniopharyngioma.

## 2 Materials and methods

### 2.1 Research object

This study was a retrospective analysis and approved by the Medical Ethics Committee of our hospital without informed consent of patients. A total of 68 patients with craniopharyngioma confirmed by surgery in our hospital from October 2018 to March 2024 were collected, including 36 males and 32 females.

### 2.2 Image acquisition

All patients underwent MR plain scan including axial T1WI, T2WI, sagittal T1WI and Coronal T2WI. The coronal T2WI scanning parameters are as follows: TR 90 ms, TE 2000 ms, slice thickness 5 mm, number of slices 12. Head advanced. After the plain scan, the patient was injected with 0.2 ml/kg Gd-DTPA through the anterior elbow vein with a high-pressure syringe immediately after the plain scan, and the infusion tube was washed with 20 ml of normal saline. After injection, enhanced axial, sagittal and coronal scans were performed.

### 2.3 Image analysis

The MRI manifestations of the tumor were evaluated on T2WI scan images. We observed the tumor’s location, and the presence of cystic degeneration, fluid levels, sediment, necrosis, calcification, supratentorial hydrocephalus, and pituitary or pituitary stalk involvement. The extent of hypothalamic involvement was also classified according to the Puget system prior to surgery. The maximum diameter (mm) of the tumor in the three planes perpendicular to each other was measured manually on the enhanced scan image, and the tumor volume was calculated according to the Toda formula V (mL)=1/2×a×b×c (a, b, c are the maximum diameter of the tumor in the three planes perpendicular to each other) (9).

### 2.4 Statistical method

All patients were divided into adult group (≥ 18 years old) and minor group(< 18 years old) according to age. The preoperative parameters, encompassing pathological subtype, gender, tumor volume, location, texture, calcification, liquid level or sediment and necrosis, necrosis, supratentorial hydrocephalus, pituitary or pituitary stalk involvement and Puget grade of hypothalamic involvement (10), were subjected to statistical comparison using the χ² test or Fisher’s exact test, as appropriate. According to the pathological type, the patients were divided into ACP group and SPCP group. Preoperative variables, including age, sex, tumor volume, anatomical location, tumor texture, presence of calcification, liquid level or sediment and necrosis, occurrence of supratentorial hydrocephalus, involvement of the pituitary gland or pituitary stalk, and Puget grade of hypothalamic involvement, were compared between the two study groups. In addition, Craniopharyngiomas were stratified by tumor location based on their relationship to the third ventricle and hypothalamic attachment (11). Patients were then simplified into two groups: the third ventricular floor invasion group and the non-third ventricular floor invasion group (12). Differences in pathological type, age, sex, tumor volume, tumor consistency, presence of calcification, liquid level or sediment and necrosis, necrosis, supratentorial hydrocephalus, pituitary or pituitary stalk involvement, and preoperative Puget hypothalamic involvement grade were compared between the two groups. The tumor volume data is expressed as the median (25%-75%). For data not conforming to a normal distribution, nonparametric tests are used to compare differences between groups. Statistical software SPSS 27.0, two-sided test, P < 0.05 was considered statistically significant.

## 3 Results

### 3.1 Clinical and pathological data

A total of 68 patients with craniopharyngioma were included, aged 4–82 years. Grouped according to age: 48 adult patients, 20 minor patients; according to the pathological type group: 44 cases of ACP, 24 cases of SPCP.

### 3.2 Pathological and imaging findings of craniopharyngioma in juveniles and adults

**Figure 1:**
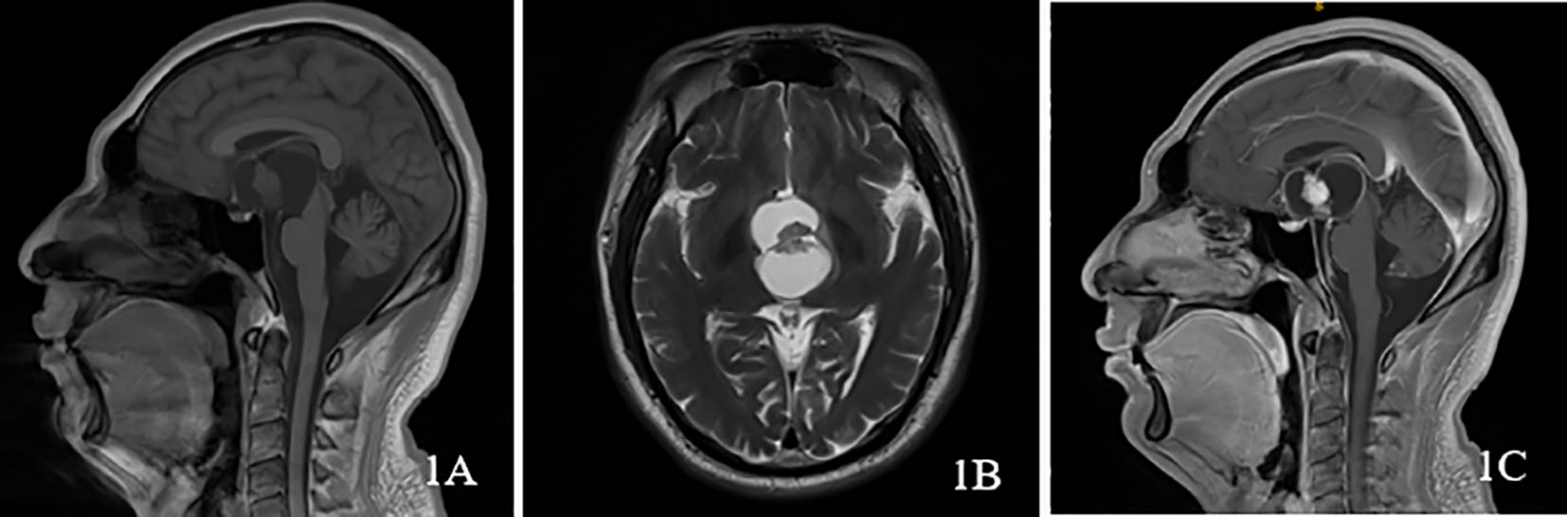
A 34-year-old male presented with SPCP. **(A)** plain scan sagittal T1WI showed suprasellar cystic solid lesions, cystic part showed low signal, solid part was equal signal; **(B)** axial T2WI showed suprasellar lesions, cystic part showed high signal, solid part was equal signal; **(C)** On the same level as **(A)**, enhanced scan showed that the solid part of the lesion was enhanced, and the cystic part was not enhanced.

No significant difference was found between the juvenile and the adult groups regarding gender, tumor volume, necrosis, supratentorial hydrocephalus, pituitary stalk involvement, or preoperative hypothalamic involvement grade. In contrast, significant differences were identified in pathological type, calcification, predilection site, and pituitary involvement (**Table 1**, **Figure 1**). Although ACP was the predominant type in both groups, its prevalence was significantly higher in juvenile patients than in adults. We observed distinct differences in tumor characteristics between the age groups. Juvenile patients predominantly presented with tumors in the intrasellar or suprasellar region, whereas adult patients typically presented with suprasellar tumors. Furthermore, juvenile patients demonstrated a significantly higher rate of calcification, while adult patients exhibited a higher probability of pituitary involvement.

**Table 1:** Clinical and imaging manifestations of craniopharyngioma patients in adult group and juvenile group.

Project	Juvenile (n=20) M (P25~ P75), n (%)	Adult (n=48) M (P25~ P75), n (%)	χ^2^	P
ACP	17 (85.0)	27 (56.3)	5.11	0.024^*^
Male	11 (55.0)	25 (78.1)	0.048	0.826
Volume (cm^3^)	10.30 (7.03~23.41)	8.29 (3.65~15.94)	–	0.098
Location			17.753	<0.001^*^
saddle	8 (40.0)	1 (2.1)		
suprasellar	8 (40.0)	29 (60.4)		
Saddle and suprasellar	4 (20.0)	18 (37.5)		
Texture				0.062
cystic	11 (55.0)	12 (25.0)		
solid	1 (5.0)	5 (10.4)		
cystic-solid	8 (40.0)	31 (64.6)		
Necrosis	8 (40.0)	28 (58.3)	1.905	0.168
Liquid level or sediment	5 (25.0)	4 (8.3)	–	0.111
Calcification	11 (55.0)	14 (29.1)	4.053	0.044^*^
Supratentorial hydrocephalus	6 (30.0)	20 (41.7)	0.814	0.367
Pituitary involvement	9 (45.0)	42 (87.5)	13.6	<0.001^*^
Pituitary stalk involvement	3 (15.0)	11 (22.9)	–	0.532
Hypothalamus involvement before Puget surgery	17 (85.0)	44 (91.7)	–	0.411

*In the table indicates a statistically significant comparison.

### 3.3 Clinical and imaging manifestations of ACP and SPCP

**Figure 2:**
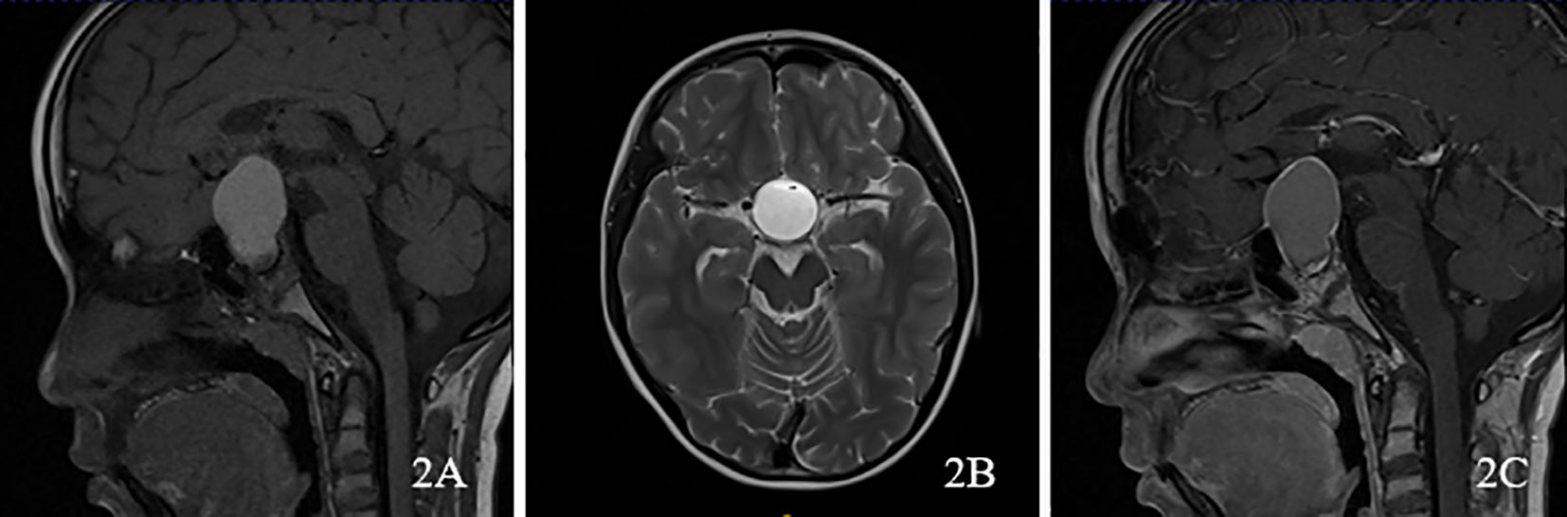
A 10-year-old male presented with ACP. **(A)** plain scan sagittal T1WI showed intrasellar and suprasellar lesions, showing high signal; **(B)** axial T2WI showed high signal; **(C)** At the same level as **(A)**, the enhanced scan showed that the edge of the lesion was enhanced.

We found no significant differences between the ACP and SPCP regarding gender, tumor volume, calcification, necrosis, supratentorial hydrocephalus, pituitary/stalk involvement, or preoperative Puget grade. There were significant differences in age, tumor texture, and the presence of intratumoral fluid level or sediment between the two tumor subtypes (**Table 2**, **Figure 2**). The age of onset was significantly lower for ACP compared to the SPCP. Both tumor subtypes can be cystic, solid, or cystic-solid. Compared with the SPCP, the ACP demonstrates a higher incidence of cystic degeneration. Furthermore, it may develop intratumoral fluid levels or sediment, features typically absent in SPCP.

**Table 2:** Clinical and imaging manifestations of ACP and SPCP.

Project		ACP (n=44) M (P25~ P75), n (%)	SPCP (n=24) M (P25~P75), n (%)	χ^2^	P
age		27 (4~64)	43 (5~82)	–	<0.001^*^
Male			16 (66.7)	2.805	0.094
Volume (cm^3^)			8.3 (3.20~12.29)	–	0.197
Location				4.763	0.092
saddle		8 (18.2)	1 (4.2)		
suprasellar		20 (45.5)	17 (70.8)		
Saddle and suprasellar		16 (36.3)	6 (25.0)		
Texture					0.037^*^
cystic		19 (43.2)	4 (16.7)		
solid		2 (4.5)	4 (16.7)		
cystic-solid		23 (52.3)	16 (66.6)		
Necrosis		23 (52.3)	13 (54.2)	0.022	0.881
Liquid level or sediment		9 (20.5)	0 (0.0)	–	0.022^*^
Calcification		19 (43.2)	6 (25.0)	2.208	0.137
Supratentorial hydrocephalus		18 (40.9)	8 (33.3)	0.377	0.539
Pituitary involvement		30 (68.2)	21 (87.5)	3.091	0.079
Pituitary stalk involvement		7 (15.9)	7 (29.2)	–	0.222
Hypothalamus involvement before Puget surgery		39 (88.6)	22 (91.6)	–	1.000

*In the table indicates a statistically significant comparison. ACP, adamantinomatous craniopharyngioma; SPCP, squamous papillary craniopharyngioma.

### 3.4 Clinical and imaging manifestations of CP with and without third ventricular floor invasion

No significant differences were observed between CP with third ventricular floor invasion and those without in terms of pathological type, sex, tumor volume, tumor consistency, presence of calcification, fluid–fluid levels or sediment, necrosis, supratentorial hydrocephalus, pituitary or pituitary stalk involvement, and preoperative Puget hypothalamic involvement grade. However, the results showed a statistically significant difference in age between the two groups: patients in the non-third ventricular floor invasion group were younger and had a wider age range than those in the third ventricular floor invasion group (**Table 3**).

**Table 3:** Clinical and imaging manifestations of craniopharyngiomas with and without third ventricular floor invasion.

Project	The third ventricular floor invasion group (n=22) M (P25~ P75), n (%)	The non-third ventricular floor invasion group (n=46) M (P25~ P75), n (%)	Z	P
ACP	14 (63.6)	30 (65.22)	-0.127	0.899
age	44.5 (10~64)	25 (4~82)	-2.249	0.025*
Male	11 (50.0)	25 (54.35)	-0.334	0.739
Volume (cm^3^)	18.50 (4.08~16.17)	9.17 (5.54~16.80)	-0.151	0.507
Texture			-0.664	0.507
cystic	8 (36.4)	15 (32.61)		
solid	3 (13.6)	3 (6.52)		
cystic-solid	11 (50)	28 (60.87)		
Necrosis	12 (54.5)	24 (52.17)	-0.182	0.856
Liquid level or sediment	1 (4.5)	8 (17.39)	-1.452	0.147
Calcification	8 (36.4)	17 (36.96)	-0.047	0.962
Supratentorial hydrocephalus	11 (50.0)	15 (32.61)	-1.118	0.264
Pituitary involvement	18 (81.8)	33 (71.74)	-0.891	0.373
Pituitary stalk involvement	4 (18.2)	36 (78.26)	-0.337	0.736
Hypothalamus involvement before Puget surgery	21 (95.5)	40 (86.96)	-1.071	0.284

*In the table indicates a statistically significant comparison.

## 4 Discussion

The most common subtype of craniopharyngiomas is the ACP, which is found in both children and adults. The embryonic theory can explain the pathogenesis of ACP. During embryogenesis, Rathke’s diverticulum, which forms at the buccopharyngeal membrane, invaginates and migrates to develop into Rathke’s pouch. Cells in Rathke’s pouch proliferate and extend to form the craniopharyngeal duct, which connects to the roof of the oral cavity and gradually regresses later. Incomplete regression of the craniopharyngeal duct may lead to abnormal proliferation of residual ectodermal cells, eventually resulting in craniopharyngioma (13). The metaplastic theory is frequently employed to illustrate the pathogenesis of PCP. Papillary craniopharyngiomas originate from the metaplasia of adenohypophyseal cells within the pituitary gland, which further form well-differentiated squamous cell nests. Although these tumors share a similar location with embryologically derived craniopharyngiomas, the key evidence supporting the metaplastic theory is that epithelial cell nests can be observed in the pituitary glands of healthy adults, especially in the glandular tissue of the pars tuberalis covering the pituitary stalk (13). The metaplastic theory is frequently employed to illustrate the pathogenesis of PCP. Papillary craniopharyngiomas originate from the metaplasia of adenohypophyseal cells within the pituitary gland, which further form well-differentiated squamous cell nests. The incidence of such epithelial nests increases with age, which also accounts for the characteristic predilection of PCP for adult patients (13).

Craniopharyngiomas have a predilection for the sellar/parasellar region and commonly exhibit local invasiveness. ACP are composed of cystic and solid elements, with calcification being a common feature. The typical imaging features of ACP have the following “90% rule”: approximately 90% of cases demonstrate cystic degeneration, about 90% exhibit calcification, and the cyst wall shows contrast enhancement in roughly 90% of patients (5, 7). In contrast, SPCP is mostly solid and have clear boundaries with surrounding tissues (14).

This study of 68 craniopharyngioma patients demonstrated no significant gender predilection. A bimodal distribution was observed in the age of the study population, with the ACP accounting for the majority of the tumor subtypes. These findings are consistent with previous literature (3, 15).

The results of this study showed a higher proportion of ACP in juvenile patients, which is consistent with previous reports (16). However, contrary to prior findings that tumors are larger in juveniles, we found no significant difference in tumor volume between the adult and juvenile groups (2). The discrepancy could be attributed to the imbalanced cohort in the previous study, which included an underrepresentation of juvenile patients, potentially introducing selection bias. Our study also found a higher incidence of tumor calcification in juvenile patients, which we attribute to the higher prevalence of the ACP in this group, a subtype itself strongly associated with calcification. In pediatric patients, the most common pathological subtype of craniopharyngioma is the adamantinomatous type, and the “90% rule” supports the findings of the present study. In addition, our study found that juvenile craniopharyngioma were more frequently located in the intrasallar or suprasellar region, whereas adult tumors were predominantly suprasellar, a finding consistent with previous studies (17). Furthermore, we found that adult patients had a higher probability of pituitary involvement than juvenile patients, whereas the two groups showed similar rates of supratentorial hydrocephalus and pituitary stalk involvement. Previous studies have shown that the structure most likely invaded by craniopharyngioma depends on its origin: the pituitary gland is most frequently involved by intrasellar tumors, the pituitary stalk by suprasellar tumors, and the floor of the third ventricle by tumors extending into the third ventricle (18). Previous studies indicated that the structure most likely invaded by craniopharyngioma depends on its origin: the pituitary gland is most frequently involved by intrasellar tumors, the pituitary stalk by suprasellar tumors, and the floor of the third ventricle by third ventricle tumors. Furthermore, analysis of MRI findings based on pathological classification revealed no significant difference in the incidence of calcification between the two tumor subtypes. This finding contrasts with some previous reports (7, 19). We propose that these discrepant findings may be attributed to the limited sample sizes and a lack of age-stratified analysis within the pathological subtypes in previous studies.

Our study compared the two pathological subtypes of craniopharyngioma and confirmed that patients with ACP present at a significantly younger age than those with the SPCP. This aligns with the established epidemiological pattern that ACP is predominantly pediatric, while SPCP is more common in adults. Furthermore, we found a higher incidence of cystic degeneration in ACP, which is consistent with previous literature (5). In ACP, aberrant excessive phospholipid synthesis in tumor cells, coupled with robust proliferation of cyst wall cells, facilitates the secretion and progressive accumulation of cyst fluid, thereby establishing an “autotrophic” cycle of cyst formation. Meanwhile, autophagic activity removes misfolded proteins and damaged organelles, releasing degradative products into the cyst lumen and further contributing to cyst expansion. Additionally, keratin degradation liberates substantial quantities of cholesterol, which constitutes approximately 70% of the cyst fluid and gives rise to the pathognomonic “machine oil–like” cystic content (20). Furthermore, our study found that intratumoral fluid levels or sediment were a characteristic feature of ACP but were absent in the SPCP. This is consistent with the known pathology of ACP, whose cysts are filled with a protein-rich, cholesterol-laden “machine oil” fluid containing numerous lipids and inflammatory mediators (16, 21). In contrast, no fluid level or sediment were observed in SPCP. This absence is consistent with the predominantly solid, less cystic nature of SPCP (3). Alternatively, the underrepresentation of SPCP cases in our cohort may have limited our ability to detect these features.

There are still some limitations in this study. First of all, our study included a small sample size, and failed to classify different pathological types of craniopharyngioma at different ages. Second, our study is a retrospective study, only the clinical data and imaging findings of previous cases were summarized. Third, our study only selected conventional MR sequences.

In summary, there is a close correlation between age at onset, pathological subtype, and MRI features of craniopharyngiomas. ACP is more common in juvenile patients, with a higher tendency toward intrasellar/suprasellar location, calcification, and cystic degeneration. In adult patients, lesions are predominantly suprasellar and more frequently accompanied by pituitary involvement. ACP show a higher cystic degeneration rate with characteristic fluid-fluid levels or sediment, which can be used for preoperative pathological differentiation. Meanwhile, invasion of the floor of the third ventricle is more commonly seen in older patients, suggesting that age may assist in evaluating the invasive growth pattern of craniopharyngiomas.

## Data Availability

The datasets presented in this article are not readily available because Data are available from the corresponding author on reasonable request. Requests to access the datasets should be directed to Weijian Wang, weijianwang520@163.com.
